# Nanoscale electromechanical properties of template-assisted hierarchical self-assembled cellulose nanofibers†
†Electronic supplementary information (ESI) available. See DOI: 10.1039/c8nr04967j


**DOI:** 10.1039/c8nr04967j

**Published:** 2018-08-30

**Authors:** Yonatan Calahorra, Anuja Datta, James Famelton, Doron Kam, Oded Shoseyov, Sohini Kar-Narayan

**Affiliations:** a Department of Materials Science & Metallurgy , University of Cambridge , 27 Charles Babbage Road , Cambridge CB3 0FS , UK . Email: sk568@cam.ac.uk ; Email: yc402@cam.ac.uk; b The Robert H. Smith Institute of Plant Science and Genetics and The Harvey M. Krueger Family Center for Nanoscience and Nanotechnology, The Robert H. Smith Faculty of Agriculture , Food and Environment , the Hebrew University of Jerusalem , P.O.B. 12 , Rehovot 76100 , Israel; c School of Applied & Interdisciplinary Sciences , Indian Association for the Cultivation of Science , 2A/2B Raja S.C. Mullick Road, Jadavpur , Kolkata 700 032 , West Bengal , India

## Abstract

Cellulose, a major constituent of our natural environment and a structured biodegradable biopolymer, has been shown to exhibit shear piezoelectricity with potential applications in energy harvesters, biomedical sensors, electro-active displays and actuators. In this regard, a high-aspect ratio nanofiber geometry is particularly attractive as flexing or bending will likely produce a larger piezoelectric response as compared to axial deformation in this material. Here we report self-assembled cellulose nanofibers (SA-CNFs) fabricated using a template-wetting process, whereby parent cellulose nanocrystals (CNCs) introduced into a nanoporous template assemble to form rod-like cellulose clusters, which then assemble into SA-CNFs. Annealed SA-CNFs were found to exhibit an anisotropic shear piezoelectric response as directly measured using non-destructive piezo-response force microscopy (ND-PFM). We interpret these results in light of the distinct hierarchical structure in our template-grown SA-CNFs as revealed by scanning electron microscopy (SEM) and high resolution transmission electron microscopy (TEM).

## Abstract 2

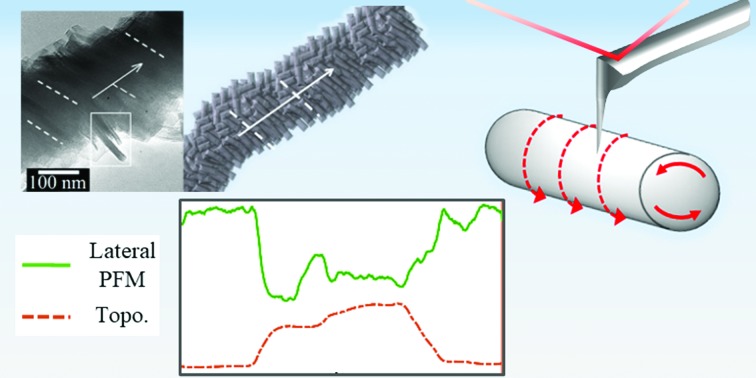
Hierarchical self-assembled cellulose nanofibers, fabricated using a template-wetting process, are shown to exhibit distinct shear piezoelectricity, paving the way towards engineered nanofibers with tailor-made electromechanical properties.

## Introduction

With the increasing global reliance on electronic devices, cheap and abundant electroactive materials are being actively sought for applications in sensors, transducers, actuators and energy harvesters. Furthermore, in order to introduce flexibility in such devices, recent work has focussed largely on the use of piezoelectric polymers that can inter-convert mechanical and electrical energy, and which are commonly durable, light weight, and can be relatively easily manufactured.^1^–^7^ Piezoelectric polymers such as PVDF (polyvinylidene fluoride), and its co-variants,^1^,^3^,^5^ odd-numbered Nylons (Nylon-11),^4^,^8^ and poly-l-lactic acid (PLLA),^6^ have been investigated for their energy harvesting properties by our group, where nanowires of these materials grown by template-wetting have been shown to exhibit superior piezoelectric properties as compared to bulk or thin films due to self-poling. Additionally, there have been several other reports on polymer-based nanogenerators, including those involving biological and biocompatible polymers.^9^–^19^ In this regard, cellulose belongs to the family of naturally occurring piezoelectric materials that has been the subject of continuous research.^9^–^14^,^16^,^20^–^26^ Nevertheless, the electromechanical properties of cellulose particularly at the nanoscale have been scarcely studied, but could hold the key to unlocking the potential of this material in piezoelectric devices.

As one of the most abundant structured bio-polymers in nature, and a major constituent of plants and woods, cellulose has been widely studied for application as optical films, coatings, pharmaceuticals, textiles and medical devices.^20^–^25^ Moreover, cellulose shows shear piezoelectricity,^13^ similar to what has been observed in many other biological polymers, such as PLLA and collagen.^6^,^11^,^12^,^14^ Fukada^13^,^26^ reported the piezoelectric coefficients of wood and verified that oriented cellulose crystallites are responsible for the observed piezoelectricity (ESI S1†) due to stress-induced orientation of dipoles, possibly stemming from the OH groups in cellulose molecules.^13^ Although the piezoelectric properties of wood in these studies were found to be weak, the origin and nature of piezoelectricity in cellulose, particularly in cellulosic nanofibrils and nanocrystals (CNCs),^27^ referred to as nano-celluloses, have continued to be periodically studied and explored. The interest in this material stems from its innate ability to form entangled porous networks for fabrication of lightweight membranes, films and hybrid nano-papers, for applications as energy efficient and biocompatible devices.^28^–^33^ Recently, thick 5–10 μm films of bacterial cellulose nanofibrils were reported for their use in piezoelectric sensors with vertical sensitivities of 5–15 pm V^–1^ in ambient conditions.^32^ This report, coupled with recent progress in using cellulose as a smart material in electro-active paper (EAPap),^25^,^34^,^35^ illustrates their unique application potential as biodegradable and disposable paper sensors, actuators and medical diagnostics. Interestingly, the piezoelectric coefficient values reported for processed CNC films,^32^,^33^ are significantly higher than those reported for wood, and are obtained by macro-scale measurements. Furthermore, the measured displacements are vertical, despite the fact that theoretically there is no *d*_*ii*_ type coefficient for wood.^13^ Nonetheless, it is probable that high level of ordering gives rise to coefficients which are averaged-out in wood, indicating that in-depth study into the cumulative interactions of aligned CNCs is required.

**Fig. 1:**
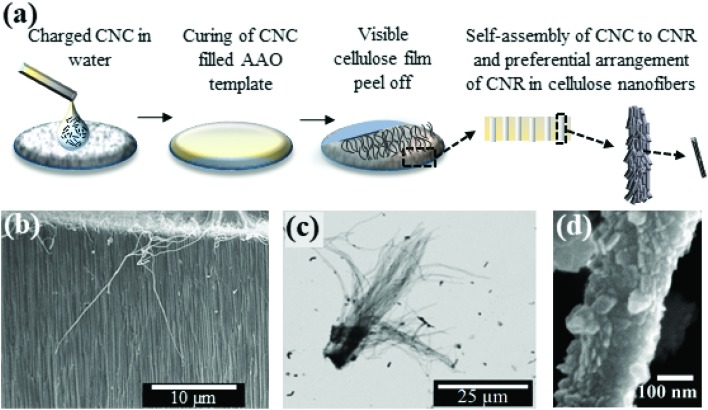
(a) Schematic of the preparation of Self-Assembled Cellulose Nano Fibers (SA-CNFs). SEM images of (b) SA-CNFs formed inside the AAO template observed in cross section, (c) separated SA-CNFs are obtained after dissolution of the template, and (d) section of an individual SA-CNF.

Self-assembly in CNCs has been a topic of discussion for last few years,^36^,^37^ and is relevant in understanding assembly in other biomaterials. Structurally, cellulose chains are linear and usually aggregation occurs *via* both intra- and intermolecular hydrogen bonds.^13^,^26^,^27^ With a strong affinity to itself and toward materials containing hydroxyls groups, CNCs can easily self-assemble in water.^27^ Rod-like CNCs with only a few nanometers of lateral dimensions have been shown to exhibit right-handed chiral twisting along the rods, while at the macro level left handed chirality is observed.^36^–^38^ The formation of hydrogen bonds at the cellulose/water interface is also observed to be highly dependent on the orientation of the CNCs in the chains, and it was argued that significant contribution from van der Waals forces contribute to the strong cohesive energy within the CNC network.^39^ In the present study, we adopt a multi-microscopy approach to investigate high-aspect ratio self-assembled cellulose nanofibers (SA-CNFs) as this geometry lends itself particularly well to flexible devices where shear forces due to bending/flexing will yield a larger piezoelectric response than that due to axial deformation. We fabricated SA-CNFs from an aqueous dispersion of CNCs using a simple template-wetting method (drop-casting),^1^,^3^ followed by a low-temperature thermal treatment process at 80 °C (Fig. 1a). Size confinement through template-based processing has been found to induce self-poling in poly(vinylidenefluoride-*co*-trifluoroethylene) (PVDF-TrFE) and Nylon-11 nanowires^3^–^5^ which is otherwise not observed in the bulk or in films, thus encouraging us to examine the effect of nano-confinement on cellulose. SA-CNFs were formed from CNCs, which above a critical aqueous concentration exhibit chiral nematic ordering as observed by transmission electron microscopy (TEM).^40^–^43^ The SA-CNFs displayed helicoidal arrangement of rod-like cellulose clusters, where the helicoidal axis follows the longitudinal axis of the pores of the anodised alumina (AAO) templates used. In a recent study, self-assembly of CNCs within a confined droplet, diminished through controlled evaporation, resulted in a concentric arrangement of the cellulose layers, rather than the commonly observed chiral-nematic structure – demonstrating the coupling between size confinement and material properties.^44^ Notably, we use the term CNF here to describe structures formed from CNCs as grown in a template-assisted bottom-up approach. This is in contrast to cellulose fibres often reported in the literature which are typically synthesised by a top-down approach, and which result in fibres or fibrils containing both crystalline and amorphous regions.^25^,^27^

In recent years, quantitative nanomechanical mapping (QNM) and piezoresponse force microscopy (PFM) have emerged as advanced scanning probe tools used to assess the mechanical and electromechanical properties of materials at the nanoscale.^36^,^45^–^47^ In peak-force QNM, a force curve is obtained at each pixel as an atomic force microscope (AFM) tip is brought into intermittent contact with the sample, which provides information about localised mechanical properties such as elasticity. Analysis of force curves is performed on the fly as the tip is scanned across the sample, resulting in the acquisition of spatially resolved mechanical properties. This imaging mode is non-destructive to both tip and sample since it directly controls the peak normal force and minimizes the lateral force on the probe. On the other hand, conventional PFM, which is used to probe mechanical deformation in response to an applied voltage bias, is a “contact-mode” AFM technique that may not be suitable for scanning nanoscale objects.^47^ Instead, we have recently built upon the traditional QNM technique to perform piezoelectric measurements of the SA-CNFs for the first time in a condition when the cantilever tip and sample are in discontinuous contact, to realise non-destructive PFM (ND-PFM)^48^ (ESI S2†), in a nano-cellulose material. In ND-PFM, an AFM tip is oscillated into “discontinuous” contact during scanning, while applying an AC bias between tip and sample and extracting the piezoelectric response for each contact point by monitoring the resulting localized deformation at the AC frequency. In this work, SA-CNFs showed higher crystallinity attributed to post-deposition thermal treatment, which resulted in enhanced mechanical properties and stability, as determined using QNM on individual SA-CNFs. ND-PFM was performed on the mechanically more stable annealed SA-CNFs, which further showed evidence of preferential arrangement within single SA-CNFs. Lateral in-plane deflections were observed only from SA-CNFs oriented parallel to the AFM cantilever, which demonstrated the presence of a selective shear piezoelectric response, and could be correlated to the hierarchical structural features in these SA-CNFs as separately revealed by scanning electron microscopy (SEM) and high resolution transmission electron microscopy (TEM).

## Results and discussion

### Growth and morphology of self-assembled cellulose nanofibers

In this work, to fabricate SA-CNFs, charged CNCs within an aqueous dispersion were drop-cast onto AAO templates facilitating self-assembly of CNCs within the nanoporous channels (see Methods section below). Following a thermal treatment process to remove the adsorbed water molecules, SEM imaging of an intentionally fractured AAO template revealed well-formed SA-CNFs, as shown in Fig. 1b. Noticeably, the SA-CNFs were found to remain attached to the residual cellulose film from the template-wetting process (schematically shown in Fig. 1a, and shown in the SEM image in Fig. 1b), which suggests strong cohesion within the cellulose molecules.^49^ Dissolution of the AAO template released the SA-CNFs which tended to agglomerate, as shown in the back-scattered SEM image in Fig. 1c. This image indicated that SA-CNFs of length ∼50 μm could be reliably obtained following the dissolution of the annealed templates. A single SA-CNF when closely observed showed rough surface texture (Fig. 1d), with a lateral dimension (∼225 nm) closely matching the nominal pore size of the AAO template. The observed surface roughness may have arisen due to the rod-like CNCs structures protruding from the surface (see schematic in Fig. 1a), which is in qualitative agreement with TEM studies shown later. However, re-dispersion of the SA-CNFs in aqueous solution caused tangling of the SA-CNFs due to adsorption of water and subsequent re-structuring (ESI S3a†). Low-temperature post-deposition thermal treatment process was found to be necessary to realise SA-CNFs of higher crystallinity and improved mechanical stability, which are essential to obtain better piezoelectric response in cellulose.^49^ The crystallinity of the SA-CNFs was determined from X-ray diffractometry (XRD) spectra of different SA-CNF samples before and after thermal treatment (ESI S4†). While all the samples did show typical cellulose Iβ peaks similar to the parent CNC sample (ESI S4b†), the degree of crystallinity as calculated form the peak intensities were found to have increased in annealed SA-CNFs with a relative crystallinity index (ESI S4a†) of 0.76, as compared to the non-annealed SA-CNFs with a relative crystallinity index of 0.48. Differential scanning calorimetry (DSC) and thermogravimetric analysis (TGA) studies further indicated strong stability in the crystalline form (ESI S4c†). QNM measurements reveal the role of the heat treatment to enhance structural integrity of the SA-CNFs (see ESI S5†).

**Fig. 2:**
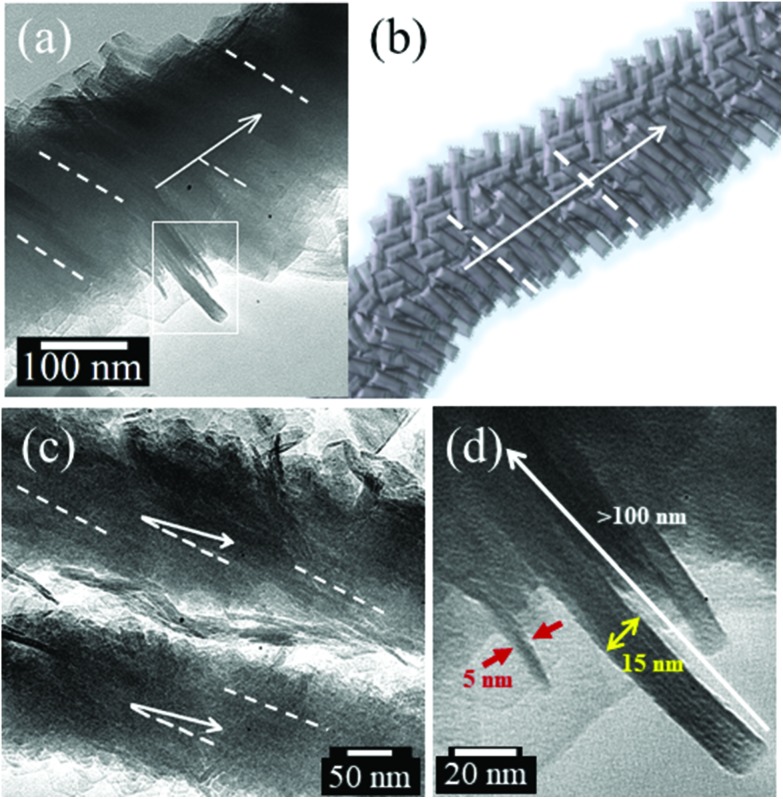
TEM images of different SA-CNFs (a) revealing helicoidal structure, presented along with (b) schematic of the SA-CNFs, (c) preferential orientation of rod-like cellulose clusters at an acute angle with respect to the SA-CNF axis. (d) Presence of individual CNC (∼5 nm in diameter) rod-like clusters having larger width between 10–20 nm and lengths >100 nm, both integrated into the SA-CNF structure.

TEM images of individual SA-CNFs revealed the presence of helicoidal structure (dashed lines *vs.* arrows in Fig. 2a). A schematic of the self-assembled structure is presented in Fig. 2b for comparison. Self-assembly of CNCs to larger rod-like clusters of width between 10–20 nm assembling to form SA-CNFs could also be observed from the TEM images in Fig. 2a and c where these clusters can be seen to preferentially orient themselves at an acute angle (between 25° and 45°) with respect to the long axis of the SA-CNF, as indicated by the arrows in the figures. Freely dispersed CNCs were also found to exhibit clustering, albeit to a smaller extent, probably due to lack of confinement (ESI Fig. S4d†). Higher resolution TEM image of protruded rod-like geometry from a SA-CNF (Fig. 2d) revealed well integrated CNCs within these rod-like clusters (ESI S3b†). High resolution imaging (Fig. 2d) shows individual CNCs (∼5 nm in width as shown in Fig. 2d, ESI Fig. S3b and S4a†), and the larger rod-like cluster with a width of ∼15 nm and length >100 nm (also see ESI S3b†). Well-ordered cellulose chains of width ∼1–2 nm for a single chain (see features within the rod-like cluster of width 15 nm shown in Fig. 2d) corresponds to that reported for cellulose Iβ.^50^,^51^ We therefore believe that a hierarchical self-assembly process was at play, involving the transformation of the chiral nematic ordered CNCs confined in the AAO nano-pore channels, *via* rod-like clusters, into SA-CNFs upon dehydration during thermal treatment. Similar rod-like structures have been recently reported from a mixture of charged gold nanoparticles (AuNPs) and CNCs.^37^ The formation of chiral rod-like structures was obtained by neutralization of the inter-CNC electrostatic forces. Interestingly, template-free self-assembly of CNC into layers resulted in chiral structures with a period of 1–3 μm.^43^ indicating that different forces are in play in this case.

In our case, we suggest that the confinement of the CNCs within the nanopore channels forces the self-assembly process to rod-like clusters, and finally to SA-CNFs, as driven by the surface energy of the template pores, which results in the morphological integrity being retained even after freeing from the template. Although the exact self-assembly mechanism is not yet clear, it might be related to the attraction due to the charged AAO template walls^52^

We suggest that the interaction between the template walls and the nanorods determines the initial configuration (nucleation) of the SA-CNFs, which is subsequently replicated to form the rest of the structure. A possible route is as follows: although the chirality of CNCs or nanorods (small CNC bundles) is right-handed,^36^,^37^,^43^ the chirality observed from CNC self-assembled films is left-handed, due to internal interactions.^37^,^43^ Interestingly, our TEM images indicate right-handed chirality in the SA-CNFs. When considering the nucleation of fibre growth on the curved wall, a chiral rod, having a twisted facet, will have a preferential alignment in an off-axis orientation compared to the nanopore axis. This alignment will conserve the right-handed chirality, unlike rod-to-rod interactions resulting in chirality inversion (see ESI in ref. 37). This off-axis, right-handed, chiral nucleation may override other effects which dominate self-assembly in the absence of a template.^43^ This mechanism is the subject of future research.

### Piezo-response force microscopy studies on individual SA-CNFs

**Fig. 3:**
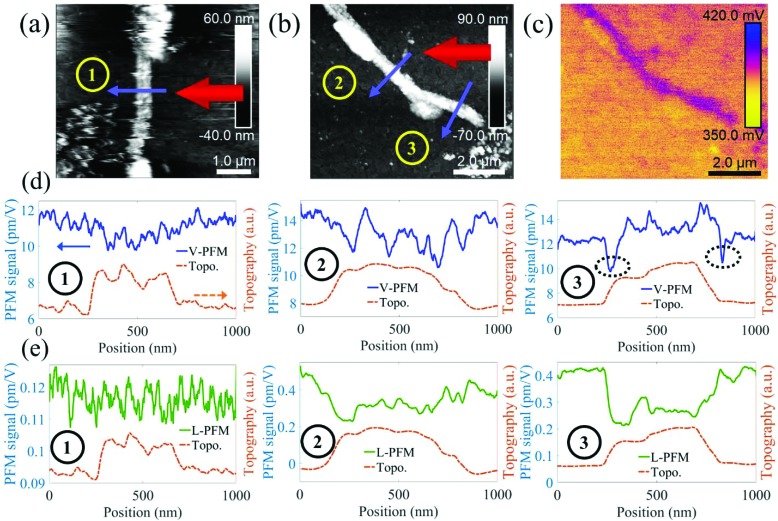
(a) QNM and (b) tapping-mode topography channels of SA-CNFs. (1, 2, 3) mark different scans acquired. The large arrow represents the cantilever and the smaller arrow shows the scan direction. (c) KPFM channel corresponding to (b). (d) Vertical ND-PFM response from SA-CNFs with varying SA-CNF-cantilever relative orientations. SA-CNF topography is shown as a guide. (e) Lateral ND-PFM response from SA-CNFs with varying SA-CNF-cantilever relative orientations. SA-CNF topography is shown as a guide. The dashed circle in (d) indicates an artefact signal resulting from weak contact between the tip and the SA-CNF (see ESI S6†).

When an electric field is applied across the sample *via* an AFM tip, a piezoelectric response is observed in the form of mechanical deformation of the sample at the point of contact. For any given deformation, there are two in-plane orientations, and one out-of-plane. The AFM cantilever picks up the out-of-plane deformations as a vertical deflection signal. The in-plane deformation in the direction transverse to the cantilever axis results in a lateral deflection signal, while the in-plane deformation in the direction parallel to the cantilever may give rise to buckling in the cantilever, thus producing a vertical deflection signal as well.^53^ Therefore, a comprehensive piezo-response analysis where in-plane deformations are expected, necessitates the rotation of the sample relative to the cantilever. For practical reasons pertaining to locating the same SA-CNF during multiple measurements, we performed PFM measurement of SA-CNFs at different orientations relative to the cantilever, rather than rotating the sample and re-measuring the same SA-CNF, under the assumption that the properties of nominally identical SA-CNFs produced from the same batch are similar. Fig. 3 shows the vertical (out-of-plane) and lateral (in-plane) ND-PFM signals obtained while scanning across three annealed SA-CNFs, which were oriented at 90° (Fig. 3a), 45° and 75° (Fig. 3b) relative to the cantilever, as shown schematically on the figures. Fig. 3c shows the Kelvin probe force microscopy (KPFM) image obtained simultaneously with Fig. 3b. The measured surface potential (400 mV) was subsequently applied while performing ND-PFM to reduce electrostatic contributions to the PFM signal.^54^Fig. 3d shows the vertical PFM signals extracted from each scan orientation, under an application of a 6 V AC excitation. The results obtained from the 4, 6, 8 V excitation correspond well with each other (see ESI Fig. S6 and S7† for more detail). Considering there is a background noise value even when measuring a non-piezoelectric conductive sample (*e.g.*, ITO),^55^ we assume the value obtained away from the SA-CNF is close to zero (with a minor electrostatic contribution). The difference of about 1–2 pm V^–1^ between SA-CNF values and the ITO, corresponds well with wood piezoelectricity,^13^ however it is smaller than reports for processed CNC films, which are 5–10 times larger,^32^,^33^ attributed to mechanical pressure and pre-poling, as mentioned above. Piezoelectric optimization was not performed in this study, and we focus our discussion on the directionality of arising piezoelectric signals in light of the self-assembly process, and the nanoscale measurements. The vertical measurement is typically considered to arise from a *d*_*ii*_ type of piezo-response, *i.e.* unidirectional electrical excitation and mechanical deformation. Nonetheless, in-plane deformations in the direction parallel to the cantilever, will also result in a vertical signal, due to buckling mode excitation,^56^ as explained above. The dashed circles in Fig. 3d indicate data obtained from poor contact between tip and sample, and can be considered an artefact due to the absence of valid force–distance curves in the raw data corresponding to these sections (see ESI S6†). Fig. 3e shows the extracted lateral PFM signals. When considering lateral cantilever displacements, it is important to note that only in-plane deformations transverse to the cantilever will result in a measured lateral signal. Interestingly, we found that there was no considerable signal (comparing the signals from the SA-CNF and the substrate) when the SA-CNF and cantilever were in the transverse configuration (scan 1). On the other hand, as the angle between the SA-CNF and the cantilever was diminished, a measureable lateral signal arose from the SA-CNFs (scans 2 and 3). This signal is much smaller than the measured vertical signal, however the calibration of the lateral signal is not straightforward and was based here on a geometrical calculation.^56^ Overall, the results indicate that there is indeed a preferential orientation of CNCs within the SA-CNFs and agrees with the observation of the oriented rod-like cellulose clusters from TEM studies (Fig. 2).

As discussed earlier, the preferential orientation is possible due to the porous template-assisted formation, as has been demonstrated for other materials.^3^,^4^,^6^,^57^ In our ND-PFM measurements, this ordering possibly gives rise to an anisotropic signal, and an apparent measurable lateral piezoelectric coefficient in certain configurations, whereas for randomly assembled CNCs, one would expect an isotropic piezoelectric response (see ESI S1†). Earlier measurements performed without surface potential minimization, have shown similar trends (however are less reliable quantitatively; see ESI Fig. S8†).

**Fig. 4:**
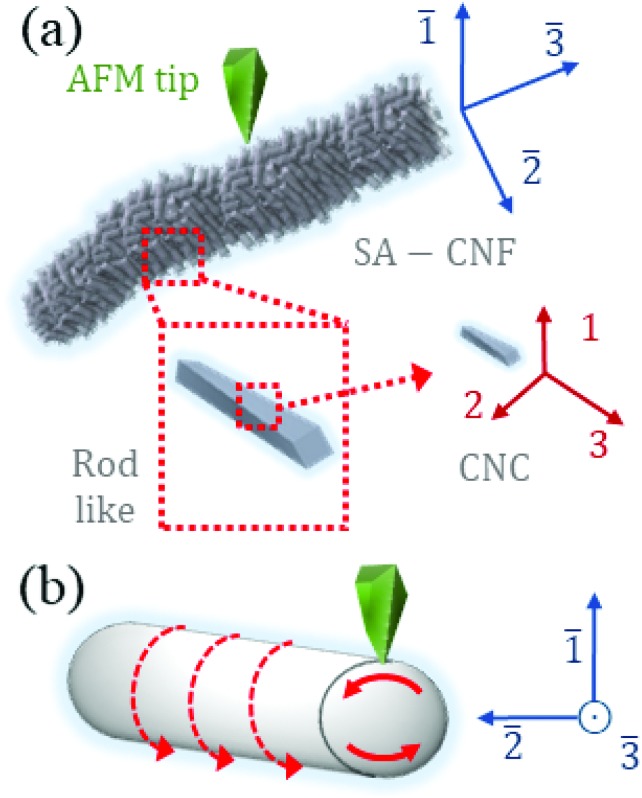
Schematic piezoelectric interactions in the hierarchically structured SA-CNF: (a) the ordering of CNCs into rod-like structures into SA-CNFs. The CNCs coordinates (red) will determine the piezoelectric response of the SA-CNF observed in the laboratory coordinates (blue, bar-symbol); (b) the influence of the helical ordering of rod-like structures in the SA-CNF may play a crucial role in giving rise to transverse piezoresponse, while attenuating axial piezoresponse.

Fig. 4a schematically shows a SA-CNF with preferential ordering of rod-like CNC clustering, and the corresponding “laboratory” measurement-coordinates (blue, marked by an upper bar), where the “material” internal coordinates dictate the actual piezoelectric behavior (red). This schematic corresponds to the TEM observation (Fig. 2), where the rod-like cellulose clusters demonstrate an ordering with relation to the SA-CNF axis. In order to discuss the ND-PFM results, we consider a simple case, where the rod-like clusters are indeed helically rotating around the main axis of the SA-CNFs; this is depicted in Fig. 4b. Upon applying the electric field from the tip through the SA-CNF, the rods on the top part of the SA-CNF (top solid arrow) will generally induce a vertical deformation and a lateral deformation with two components (axial and transvers in the SA-CNF coordinates). The rods on the bottom part (bottom solid arrow), forming a helical mirror image, will however form an opposite reaction in the rod coordinate system, which may result in the cancellation of the vertical and axial deformation and enhancement of the transversal one, due to the helical arrangement. This could explain the observed shift of signal strength from dominantly vertical (actually an in-plane transverse signal coupled to cantilever buckling) and negligible lateral signal in the 90° case (scan 1), to an additional significant lateral signal in the 75° case (scan 3). Furthermore, the tip-CNF geometry induces inhomogeneous electric field in the material which may give rise to further complexity in the piezoelectric deformation.

### Finite element simulations of PFM measurements on SA-CNFs

In order to examine this hypothesis, we performed a set of COMSOL simulations corresponding to PFM measurements. We have used similar simulations previously to examine inhomogeneous PFM field effects for PLLA as well as for GaAs semiconductor nanowires.^6^,^59^ These simulations are based on two simplifying assumptions: (i) the SA-CNFs are comprised of tube-like aligned cellulose with a dominant crystalline arrangement, chirally shifted compared to the main axis – as supported by TEM images (Fig. 2); (ii) these structures collapse to a bilayer-like structure, with a thickness of about 30–50 nm, and width of hundreds of nanometres – supported by AFM topography (see ESI S9†). Therefore, the simulation effectively comprises of two layers of cellulose, where the top layer is rotated about the normal axis (2-axis of the piezoelectric matrix) by *θ* (see Fig. 2), and the bottom layer is similarly rotated by *θ* about the normal, and additionally by 180° about the *z*-axis (3-axis of the piezoelectric matrix). This would correspond to flattening the wire depicted in Fig. 4b. Two types of piezoelectric coupling matrices were considered: a degenerate one – “wood”, and a nondegenerate monoclinic-type matrix “sucrose” (inspired by ref. 58, see ESI S10† for details of the matrices used). Since the bottom contact is treated as mechanically clamping the structure, a thickness asymmetry was introduced to intensify the rotated bilayer effect, such that the top and bottom layer thicknesses are 5 and 25 nm, correspondingly.

**Fig. 5:**
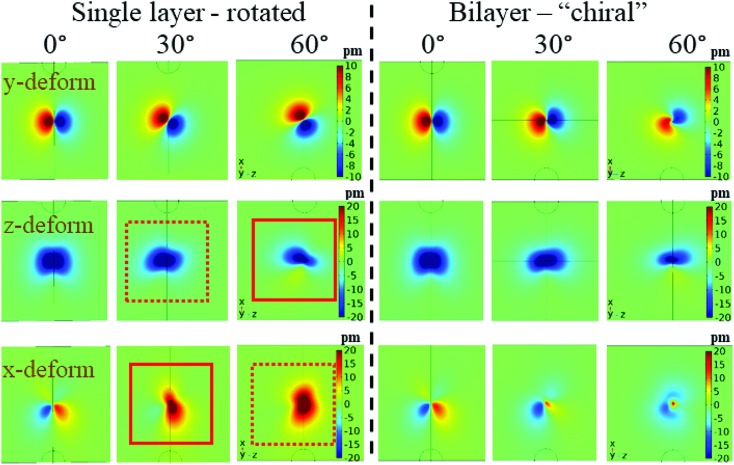
Top view (down the *y*-axis) of the deformation maps of the top (free) surface of the cellulose single (left-hand side) and bilayer (right-hand side) structures. Each structure is examined in three orientations 0° (no chirality/rotation), 30° and 60° chirality/rotation. The deformations are sorted according to those in *y*-axis, *z*-axis and *x*-axis in first, second and third rows, correspondingly. Notice that these do not directly correspond to measured PFM signals.

Fig. 5 shows important simulation results, demonstrating the bilayer model influence. It is important to note that it is not straightforward to tie the simulation results with PFM signals. For example, the vertical deformation (*y*-axis in this case), although distinctive, will probably not result in actual vertical PFM signal. This is due to the fact that the deformation has a different sign on opposite sides of the tip, and it is more likely to result in an effectively measured lateral signal (or buckling signal, which may be disguised as a vertical signal depending on tip orientation). The simulation results raise several interesting points: first of all, when using the degenerate “wood” piezoelectric matrix (see ESI S10†), only shear deformation is seen (data not shown). While this is to be expected, it bears consequences for previously reported results, where *d*_33_ type excitations were interpreted as *d*_25_ values. Indeed, in light of this, a paradigm shift is required when analysing piezoelectricity of highly oriented cellulose films.

Our simulations indicate that the non-degenerate matrix is better suited to model the experimental results. Fig. 5 shows the *x*,*y*,*z* deformation maps of the top surface when the tip is exactly at the centre of the sample. Simulations were performed for *θ* = 0°, 30° and 60°, and for the “chiral” bilayer structure as well as a simple layer (corresponding to the top layer) with the cumulative thickness. The main result of the simulation is that the bilayer structure acts to prevent the deformation pattern to follow that of a single layer, and directs the response for certain axes: the vertical deformation (top row) is somewhat pinned to the two plane halves of *z*-axis, giving rise to a lateral PFM response in that direction. The *z*-axis deformation (middle row) is relatively maintained, although in an opposite trend to the vertically-generated lateral signal. The *x*-axis deformation is diminished compared to the single layer case. Furthermore, when considering only one layer, the rotation of the layer basically causes the transfer of *z*-axis deformation to *x*-axis. Indeed, there is a rotation and mirror symmetry between the *z*(*x*) signals in 30°, and those of the *x*(*z*) signals in 60°, as expected. This is marked by the solid and dashed squares. This duality is removed in the chiral case, thus demonstrating the constructive/destructive cumulative effects of the two layers. This trend supports our hypothesis of preferential orientations in the SA-CNFs PFM response due to chirality. Future work will take into account the internal structure of the SA-CNFs, which probably induces additional degrees of freedom, compared to a single domain as examined here.

## Conclusions

In conclusion, we report for the first time the preparation of SA-CNFs, following a traditional, scalable AAO template-wetting method.^1^,^3^–^6^ A remarkable two-stage hierarchical self-assembly process was observed in which constituent CNCs first form rod-like clusters of larger dimensions which finally assemble into SA-CNFs. The presence of a helicoidal structure in a SA-CNF was clearly visualized using TEM, resulting from the locking of the chiral nematic phase of the constituent CNCs. TEM images suggest the SA-CNFs exhibit right-handed chirality, as opposed to left-handed chirality in self-assembled CNC films, and we attribute this finding to the interaction of the CNCs with the template walls. ND-PFM measurements on individual annealed SA-CNFs were found to have a predominant lateral piezo-response in the axial direction, which we attribute to their chirality. Finite element simulations were found to corroborate this explanation. Given that cellulose is already an attractive material for biodegradable and wearable sensors,^9^,^25^,^28^–^31^,^33^,^34^ the facile fabrication approach for SA-CNFs presented here, as well as the multi-microscopy approach adopted to understand their fundamental structure and piezoelectricity, may pave the way for studying self-assembly in other piezoelectric chiral phase biomaterials. Our studies therefore offer insight into possible routes towards engineering nanofibers with tailor-made electromechanical properties, by controlling the way in which chiral-nematic liquid crystals self-assemble *via* template-assisted nano-confinement.

## Materials and methods

### Materials preparation

#### Extraction and stabilization of CNCs as aqueous suspension

Cellulose nanocrystals (CNCs) were extracted according to the procedure by Beck-Candanedo *et al.* 2005.^60^ The source material for the suspension was bleached, softwood Kraft pulp (TEMBEC). TEMBEC board was cut into strips and dried overnight at 50 °C. The strips were mixed with sulfuric acid and stirred at 45 °C for 45 min, at the ratio of 1 : 17.5, 40 g TEMBEC with 700 mL sulfuric acid (64%). Then, the sample was diluted 10 times in cold double distilled water (DDW), and the mixture was left standing for 1 hour. The acidic upper phase was decanted and discarded, and three wash cycles were performed on the bottom phase according to the following sequence per cycle: the material was centrifuged (20 °C, 6000 rpm, 10 min), the supernatant was discarded, and the pellet was rinsed with DDW. The pellet from the final cycle was collected with the addition of DDW, and dialyzed against DDW until the pH of the suspensions stabilized. Finally, the suspension was sonicated (Q500 Qsonica; 6 mm probe) on ice to avoid overheating, until the suspension appeared uniform (15 kJ g^–1^). The sonicated suspensions were filtered (Whatman 541) and toluene (100 μL L^–1^) was added to the suspensions to avoid bacterial growth.

#### Preparation of cellulose nanofibers

SA-CNFs were prepared by template-based drop-cast wetting method from an aqueous dispersion of CNCs. In the process, charged CNC dispersion^60^ (1.25%) is pooled on top of the anodised aluminium oxide (AAO) porous template (Anapore, Whatman) with nominal pore diameters of ∼250 nm and of thickness 60 μm. The suspension pool of the CNC dispersion is then allowed to infiltrate the pores by gravity. The template was then left under ambient conditions, allowing the evaporation of water and self-assembly of CNCs within the pore channels. Post-preperation heat treatment of the infiltrated template at approximately 80 °C was carried out for 30 minutes to remove bound water and to facilitate the increase in the degree of bonding between CNCs, a technique earlier adopted by Jiang *et al.* for self-assembly of CNC ultra-thin films.^49^

SA-CNFs are released from the template by dissolving the AAO template in 3.2 molar potassium hydroxide aqueous solution, followed by repeated washing with deionised water, and centrifugation to neutralise and isolate the SA-CNFs for further characterisation.

### Characterisation & measurement techniques

High resolution transmission electron microscopy (HR-TEM) of CNC and SA-CNFs were acquired using a FEI Tecnai T12 G2 Spirit Cryo-TEM and FET Tecnai T20 STEM equipped with Gatan Imaging Filter, respectively. For cryo-TEM of CNCs, 0.1 wt% sample were placed on a carbon grid and vitrified (rapid freezing) in liquid ethane using a Vitrobot Mark IV (FEI Company) and cryogenically transferred in liquid nitrogen to the cryo-TEM holder, which was then inserted into the microscope (FEI Tecnai T12 G2 Spirit). The temperature of the sample was maintained at approximately –175 °C to prevent crystallization of ice. The goal of this method is to directly observe the nanocrystals as they exist in aqueous suspension. For standard room temperature TEM of SA-CNFs, released nanofibers were drop cast on copper grids and imaged alternatively between 100–120 kV at 3 spot-size to avoid destruction of the SA-CNFs by electron beam.

AFM measurements were carried out using a Bruker multimode 8 (with Nanoscope V controller). Several scanning modes were used within this study: (1) tapping mode using an MESP-RC V2 (Bruker) tip for topographic measurements; (2) quantitative nanomechanical mapping (QNM), where the cantilever periodically indents the samples and mechanical data is extracted from the measured force curves. QNM measurements were carried out with a DDESP-V2 tip, where deflection sensitivity was calibrated using a sapphire standard, and elastic modulus was then calibrated on a polystyrene film standard of known elastic modulus (2.7 GPa); (3) Kelvin probe force microscopy (KPFM), where the surface potential of the sample is measured in a second, elevated cantilever pass, following a tapping mode pass. This value is then applied to the system when performing PFM; (4) PFM measurements were performed by adapting the QNM mode to yield PFM data, in a non-destructive intermittent contact mode (ND-PFM).^48^ Calibration of the ND-PFM signal was carried out by poling and measuring a 100 nm poly(vinylidene fluoride-trifluoroethylene) (PVDF-TrFE) film, where a value for *d*_33_ coefficient of 30 pm V^–1^ was used. An MESP-RC V2 tip was used for ND-PFM scanning atop the dispersed NFs, which were lying on a conducting indium tin oxide (ITO) substrate. An alternating voltage of amplitude ∼6–8 V at a frequency 125 kHz was applied between the sample and the tip. Different tips were used for measurements and calibration, however, all were MESP-RC-V2.

### COMSOL simulations

COMSOL (version 5.3a) simulations were performed using a piezoelectric multiphysics module. The layers of cellulose were modelled clamped to a surface which was also biased, and touched by a non-mechanical (air) sphere which was grounded. The entire structure was considered to be inside air with grounded boundaries. See ref. 6 for more details. To realise cellulose as a piezoelectric material, PVDF was taken as a starting point and the strain-charge coupling matrix edited for “wood” and “sucrose” (ESI S10†). No other parameters were changed, and the matrix elements used were a rough approximation, therefore the results should be treated as a qualitative guide to the effect of the bilayer piezoelectric response.

## Data Availability

Supporting data for this paper are available at the DSpace@Cambridge data repository (https://doi.org/10.17863/CAM.25763).

## Author contributions

A. D. & Y. C. contributed equally to the work and share joint first-authorship. A. D. & S. K.-N. designed and guided the experimental work. D. K. & O. S. supplied the CNC material. J. F. & A. D. jointly fabricated the nanofibers and carried out structure and morphology studies. J. F. & Y. C. performed the AFM, QNM and ND-PFM measurements. Y. C. analysed the PFM data and performed finite element simulations. A. D., Y. C. and S. K.-N. co-wrote the paper. All authors discussed the results and commented on the paper, and have given their approval to the final version.

## Conflicts of interest

There are no conflicts to declare.

## Supplementary Material

Supplementary information
