# Supplementary material for: Nanoscale electromechanical properties of template-assisted hierarchical self-assembled cellulose nanofibers
Source: Nanoscale. 2018 Aug 30;10(35):16812–21. doi: 10.1039/c8nr04967j (PMC6137605; doi:10.1039/c8nr04967j)
Supplement: Supplementary file 1 [file NR-010-C8NR04967J-s001.pdf]

Electronic Supplementary Material (ESI) for Nanoscale.
This journal is © The Royal Society of Chemistry 2018

Supporting Information

Nanoscale Electromechanical Properties of Template-Assisted Hierarchical Self-Assembled Cellulose Nanofibers

Yonatan Calahorra[1#&], *Anuja Datta*[1&], *James Famelton*[1], *Doron Kam*[2], *Oded Shoseyov*[2], *& Sohini Kar-Narayan*[1*]

[1]Department of Materials Science & Metallurgy, University of Cambridge, 27 Charles Babbage Road, Cambridge CB3 0FS, UK.
[2]The Robert H. Smith Institute of Plant Science and Genetics. The Robert H. Smith Faculty of Agriculture, Food and Environment, the Hebrew University of Jerusalem, P.O.B. 12 Rehovot 76100, Israel.

[*]Email: sk568@cam.ac.uk
[#]Email: yc402@cam.ac.uk
[&]A.D. and Y.C. share equal contribution of authorship

S1. Shear Piezoelectric component in cellulose

Cellulose can adopt several crystalline forms,[1] the most common form, found in wood and other higher plants, is cellulose Iβ which has monoclinic crystal structure, with space group symmetry $P2_1$.[1] This symmetry leads to the following form of the piezoelectric strain tensor (using Voigt notation):

$$d_{ij} = \begin{vmatrix} 0 & 0 & 0 & d_{14} & d_{15} & 0 \\ 0 & 0 & 0 & d_{24} & d_{25} & 0 \\ d_{31} & d_{32} & d_{33} & 0 & 0 & d_{36} \end{vmatrix}$$

Where *i* is the polarisation axis and *j* is the stress axis, with *j* = 4, 5 and 6 referring to a shear stress in the plane normal to the 1, 2 and 3 axes respectively. The 3 direction is parallel to the longitudinal axis of the cellulose molecules. This is the case for a single crystallite, however if there is a collection of crystallites, all orientated in the 3-axis but randomly orientated in the 1 and 2-axes then the tensor simplifies further to;

$$d_{ij} = \begin{vmatrix} 0 & 0 & 0 & d_{14} & 0 & 0 \\ 0 & 0 & 0 & 0 & d_{25} & 0 \\ 0 & 0 & 0 & 0 & 0 & 0 \end{vmatrix}$$

where $d_{14} = -d_{25}$.[2] This is the form of the tensor which has been observed experimentally for wood.[3] The piezoelectric response of cellulose depends on the degree of crystallinity and on the

crystallographic alignment. Recent work [4] has concerned the production of highly crystalline films of cellulose, by the extraction of cellulose from wood.

Cellulose has a hierarchical structure, with elementary fibrils formed of 36 cellulose chains, around 6 of which clump together to form a microfibril, which is the main structural element in plant cell walls.[1] The elementary fibrils have both amorphous and crystalline regions. Acid hydrolysis of the elementary fibrils can be used to preferentially hydrolyse the amorphous regions, releasing the more stable crystalline regions into an aqueous dispersion. These crystallites left after

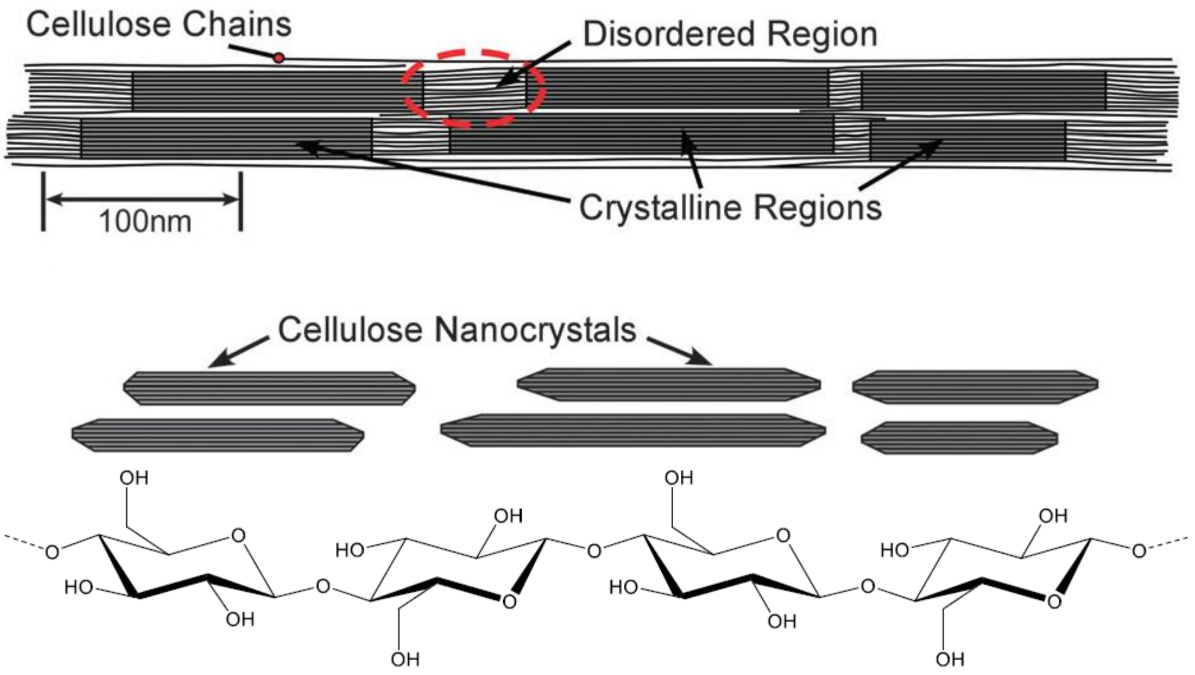

Figure S1. Hierarchical structure of cellulose, a) Schematic of a cellulose microfibril, reproduced from ref. (1) b) Cellulose nanocrystals produced by acid hydrolysis of microfibril, reproduced from ref. (1) c) Structure of single cellulose chain, formed from D-glucopyranose monomers (glucose) linked by β 1→4 glycosidic bonds.[1] Copyright, Royal Society of Chemistry, 2011.

hydrolysis are known as cellulose nanocrystals (CNCs) and have an elongated morphology with typical geometries of 3-5 nm cross-sectional diameter and 50-500 nm in length. Frka-Petesic et al. have shown that CNCs exhibit a relatively large permanent dipole moment of $1.5 \pm 0.15 \times 10^{-26}$ C m along the length of the CNCs,[5] which they attribute to the combination of the glucosyl monomer dipole and the non-centrosymmetric symmetry of the cellulose crystal.

S2. Non Destructive Intermittent contact PFM (ND-PFM)

ND-PFM is a new technique which is a variant of force-volume mode (implemented on QNM by *Bruker)* which utilizes the intermittent mechanical mode of an AFM to take piezoelectric measurements without constant sample-tip contact, as is done with conventional PFM. This allows measurement of polymer NWs which prevents destruction of the polymer NWs when imaged in

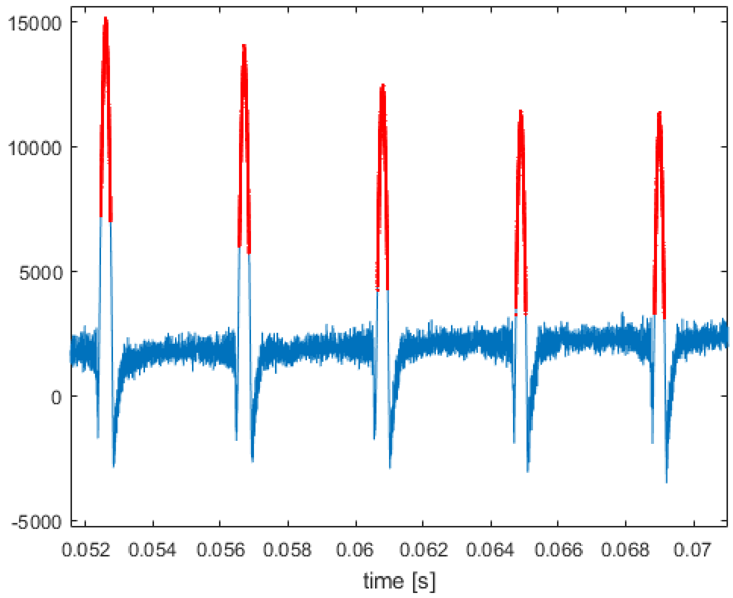

Figure S2. Part of deflection signal for a single PF-QNM scan. The region highlighted in red indicates the part of the signal that is fed into the lock in amplifier.

contact mode. In PFM a conducting tip is held in contact with the sample and an alternating voltage of known frequency is applied across the tip and sample holder. A lock-in amplifier is then used to extract signals from the cantilever deflection which occur at the same frequency as the applied voltage. The deflections are caused by the inverse piezoelectric effect whereby the material is strained in response to an electric field. ND-PFM measurements are taken in QNM mode where the tip and sample are in intermittent contact. The signal is then later analysed using a "virtual lock-in amplifier" and only the time periods during the scan that the tip and sample were in contact are analysed (Figure S2).

The lock-in amplifier outputs an in-phase response (X) and quadrature response (Y) which are related to the amplitude (A) and phase (ϕ) of the response by the following relations;[7]

$$A = (X^2 + Y^2)^{1/2} \quad (1)$$

$$\phi = \arctan(X/Y) \quad (2)$$

Results presented for cellulose are in the form of in-phase and quadrature measurements where the quadrature signal has been minimized by arbitrarily altering the lock-in reference phase,[6] such that the signal response is contained within the in-phase signal. Calibration of the ND-PFM signal was carried out by scanning a reference sample of known piezoelectric response. Periodically poled lithium niobate was used from this purpose, with d_{33} = 7.5 pmV^{-1}. The magnitude of the in-phase response was measured at either side of a ferroelectric boundary.

In this kind of measurement there are parasitic effects such as electrostatic contributions to the signal and background noise.[6,7] No particular operations were taken to minimize the

electrostatic contribution, and we consider the vertical signal to be highly affected by it. The lateral signal should be less affected due to the nature of this interaction. The lateral signal was calibrated from the vertical response of the reference sample based on the work of Peter et al.[8] ND-PFM measurements were carried out with a MESP-RCV2 tip and with the SA-CNFs dispersed on a conducting indium tin oxide (ITO) substrate. An alternating voltage of amplitude 4.0 V and frequency 125 kHz was applied between sample and tip.

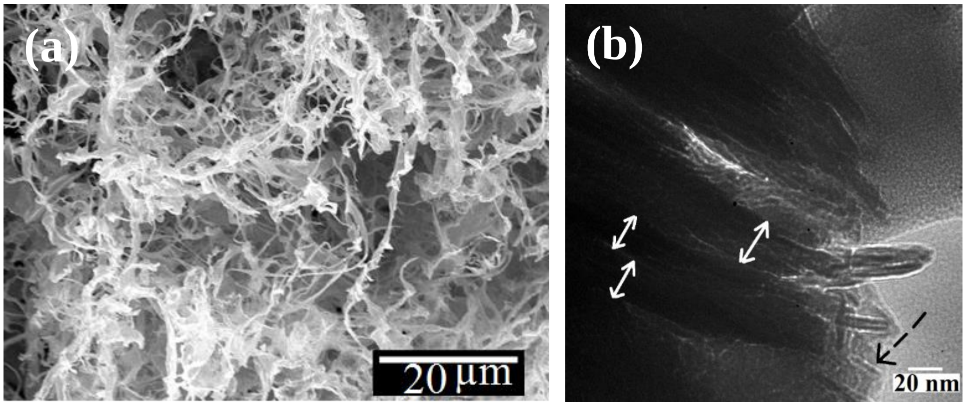

Figure S3. (a) Tangled SA-CNFs obtained after dissolving the uncured CNC filled template. (b) TEM image revealing the self -assembly of CNCs (shown with dotted arrow) to rod-like cellulose clusters (shown by double arrowheads) within a SA-CNF.

S4. Morphology and Structural analysis of CNC and SA-CNF

The X-ray diffractometry (XRD) spectra of different SA-CNF samples before and after annealing are shown in Figure S4a. While all the samples did show typical cellulose Iβ peaks similar to the parent CNC sample (Figure S4b), the degree of crystallinity as calculated form the peak intensities were found to have increased in annealed SA-CNFs with a relative crystallinity index of 0.76, as compared to the non-annealed SA-CNFs with a relative crystallinity index of 0.48. Differential scanning calorimetry (DSC) measurements on the annealed SA-CNFs (Figure 3b) indicated strong stability in the crystalline form, as the SA-CNFs did not melt upon heating, but instead degraded at around 320-390 °C, as also observed for bulk-extracted cellulose.[48] The sharp endotherm due to this degradation was readily observed for the annealed SA-CNFs with higher crystallinity (Figure S4a). The latent heat from this peak was 6.0 kJ kg^{-1} which is lower in comparison to the literature value of 553 kJ Kg^{-1} for bulk cellulose extracted from natural cotton.[48] As a result of large surface-to-volume ratio in SA-CNFs, the thermal stability differs significantly as compared to the bulk, leading to a suppression of the melting point as well as a decrease in latent heat for fusion.[49]

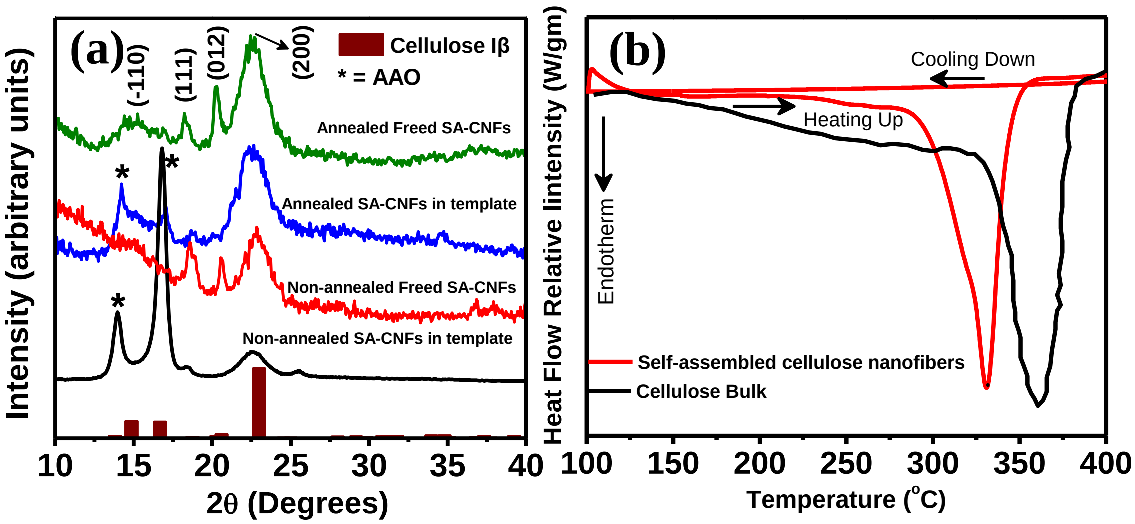

Figure S4a. (a) XRD spectra of SA-CNFs before and after annealing in comparison with standard cellulose Iβ pattern. (b) DSC spectra of SA-CNFs with reference to the bulk cellulose spectra as extracted from Hirata et al.[48]

The XRD profile of the CNC sample (Figure S4b) exhibited the known characteristic peaks; a sharp peak around 2θ = 22.5°, two not very well-defined peaks at 2θ =~14.8°,~16.5°, and one small peak around 2θ = 34.6°. The four peaks are assigned to planes $(1\bar{1}0)$, (110), (200), and (004)

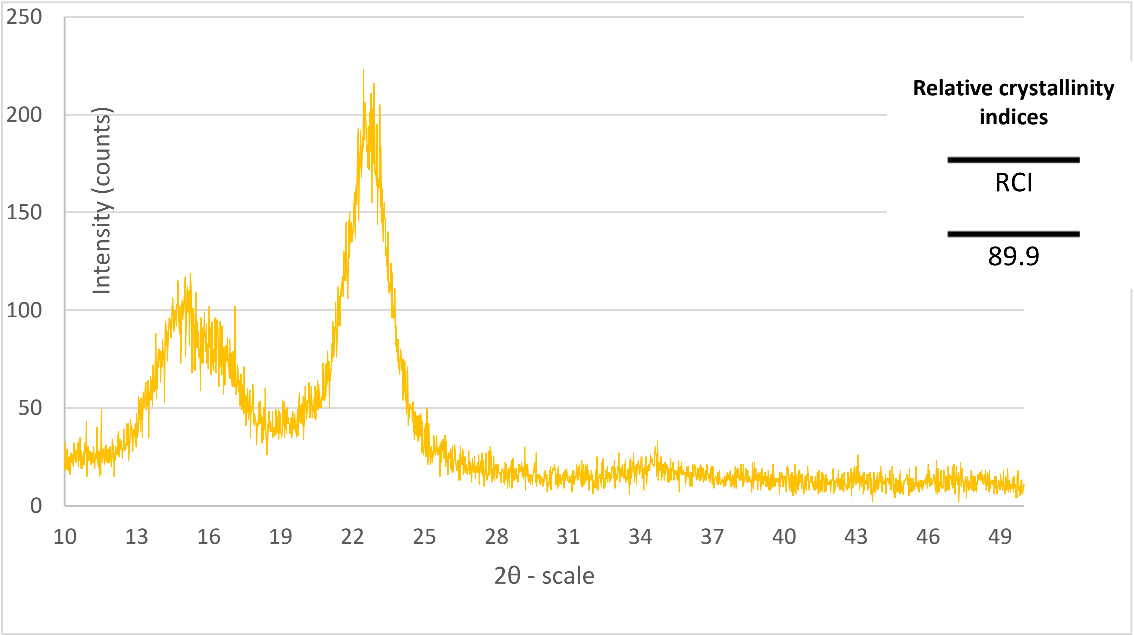

Figure S4b. XRD spectrum of parent CNCs.

which are thought to represent typical cellulose Iβ structure.[7-15] The relative crystallinity indices (RCIs) were calculated as: $(I_{200}-I_{min})/I_{200} \times 100$, where, I_{200}, is the intensity above baseline at 2θ = 22.5°, and I_min is the minimum intensity above baseline near 2θ = 18°, between (200) and

(110)/(11$\bar{0}$) peaks.[16-18] To insure objective quantitation of RCI, additional calculations were performed as described above on diffractograms that had been smoothed using the least-squares

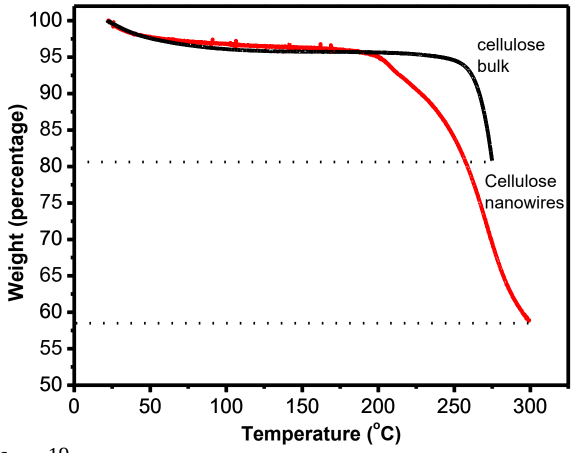

method of Savitzky and Golay.[19]

Fig. S4c TGA graphs showing percentage weight loss for cellulose bulk and nanowires when heated upto 300 °C.

Preliminary measurements of the thermal and thermo-oxidative stability of the cellulose nanowires and bulk were made using TGA and the thermograms are shown in Fig. S4d. The onset of degradation in nitrogen occurs at ca. 260 °C for the bulk but around 200 °C for the nanowires at 5°C/min. heating rate. However, cellulose nanowires showshigher temperature stability as compared to the bulk and the final degradation occurs around 300 °C, while the bulk degrades at ca. 275 °C. Cellulose bulk shows a weight loss of around 20 % when heated while cellulose nanowires show higher weight loss of around 44 % when heated at the same temperature. This could be related to the more adsorption of water for cellulose nanowires at room temperature as compared to the bulk. Nanowires having higher surface to volume ratios are more likely to adsorb more water than the bulk, which when heated are released and imparts higher weight loss.

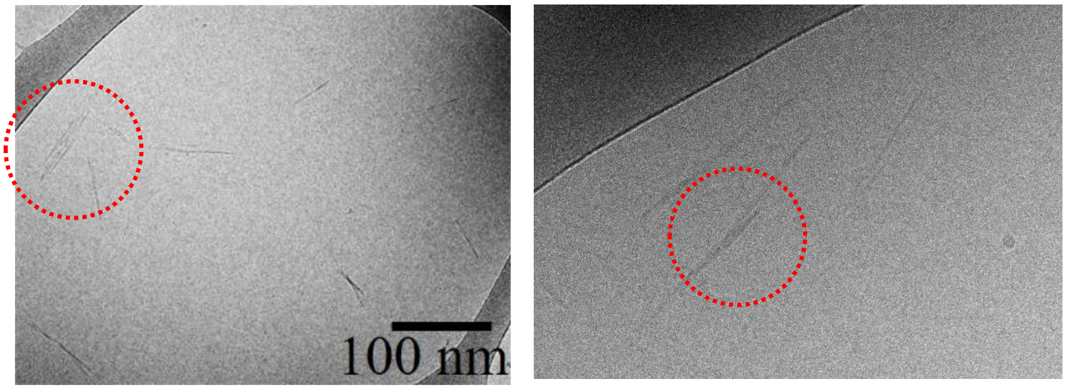

Figure S4d. TEM image of parent CNCs, dashed circles shows close packed CNCs. Scale bar applies to both images.

S5. Quantitative nanomechanical mapping (QNM) of individual SA-CNFs

A wide range of values is often quoted for the Young's modulus of cellulose in the literature which are highly dependent on the degree of fibre alignment. Moon et al.[1] quoted a value of 120-138 GPa for the axial elastic modulus of cellulose Iβ from XRD experiments from several sources,[21-23] and a value of 18-50 GPa for transverse modulus of CNCs from AFM indentation experiments.[24] CNC films have been produced by Hoeger et al.,[25] and Jiang et al.,[26] with transverse moduli of 8.3 ± 0.9 GPa and 8.3 GPa respectively. They also observed an increase in modulus up to 9.7 GPa following heating at 80 °C (17% enhancement after thermal treatment),[26] which was attributed to the formation of an enhanced intermolecular hydrogen bonding network between CNCs upon removal of bound water. Hence our SA-CNFs can be expected to show lower moduli than bulk cellulose-based materials, as the latter have chains which cross between crystalline regions, while our SA-CNFs are formed from self-assembled rod-like CNC clusters with no covalent cross-linking between them. Since the bonding between CNCs are primarily intermolecular, this will lead to an intrinsically lower modulus than for covalent bonding. Thus, it is expected that the SA-CNFs will have an elastic modulus more comparable to that of CNC films. QNM imaging of the SA-CNFs are shown in Figure S5a. In QNM, the mechanical properties of the sample are presented in real-time, through instantaneous analysis of periodical indentation curves (force curves) performed while raster scanning the cantilever over the sample. Elastic moduli of 1.6 ± 0.6 GPa and 0.18 ± 0.05 GPa were respectively obtained for annealed and non-annealed SA-CNFs that were freed from the template and dispersed onto an indium tin oxide (ITO) coated glass substrate, (the modulus scales are different for better visibility, see Figure S5b). We note that the modulus was extracted by fitting the data to an infinite plane indentation, which might have a fundamentally different mechanical response than cylinder indentation. Nonetheless, the

qualitative distinctions between the materials before/after thermal treatment are still valid. Furthermore, based on previous preliminary simulations of this problem regarding the exact geometry of indentation, the fitted values are not expected to deviate significantly from the true value of the modulus. It is therefore clear that the SA-CNF modulus (hence the stiffness) had increased due to the thermal treatment process, which might be due to stronger bonding between constituent CNCs as a result of the release of intra-molecular water, as previously observed by Jiang et al. [26] These values are lower than those quoted in the literature for CNC films and suggest there may be weaker bonding between the CNCs in the SA-CNFs than in a CNC film, or may be due to the effect of measurement geometry. The images show clear evidence of "tip-doubling", which add uncertainty to the measured values. The annealed SA-CNFs were chosen for subsequent PFM measurements, as these were found to be mechanically more robust when probed using an AFM tip.

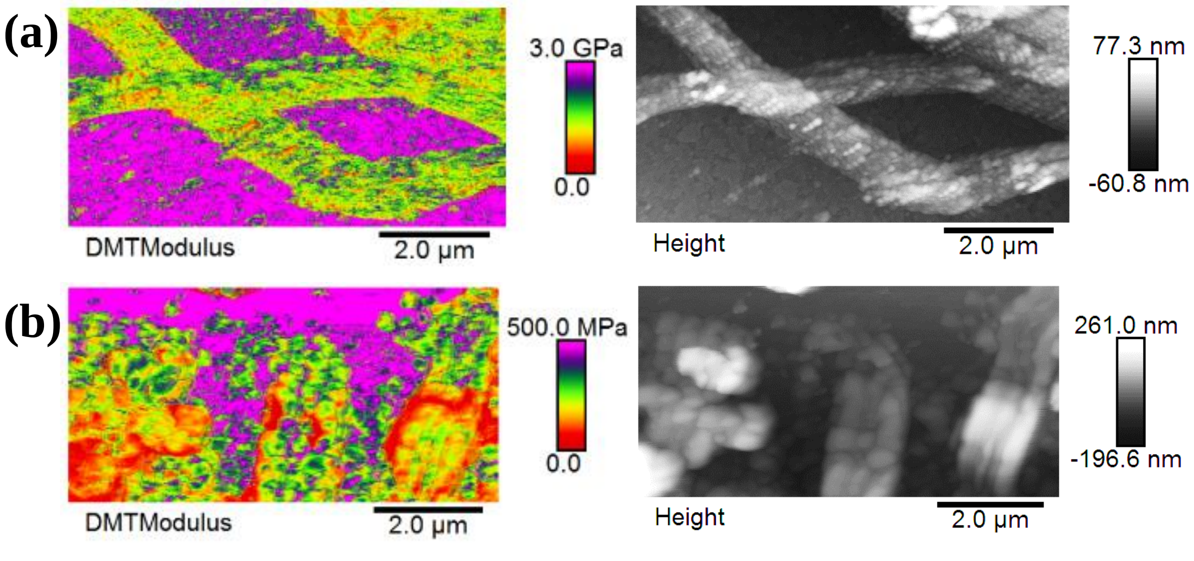

Figure S5a. (a) DMT modulus and height profile for cured freed SA-CNFs. (b) DMT modulus and height profile for non-thermally treated freed SA-CNFs. The difference in scales between DMT images and that the ITO substrate are at a much higher modulus and is thus saturated at this scale.

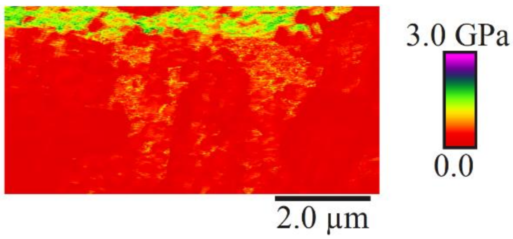

Figure S5b. Scales of mechanical measurements. The elastic modulus channel shown in Figure S5a, at a 0-3 GPa scale, identical to Figure S5a in the main manuscript.

S6. Raw deflection data for PFM

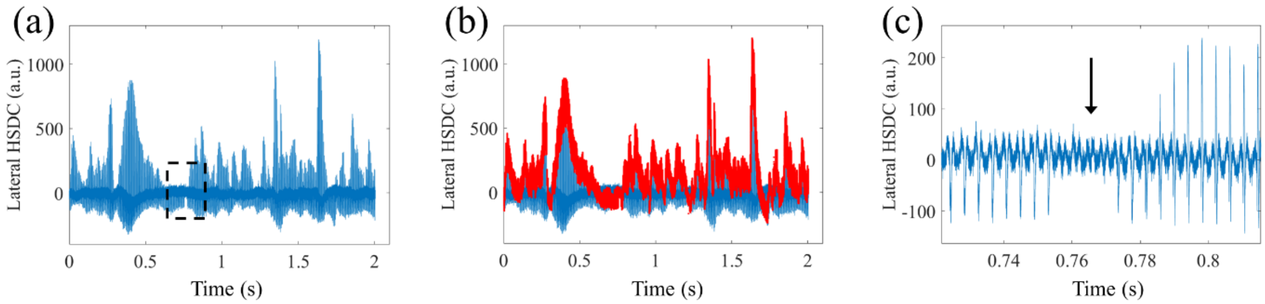

Figure S6. Raw lateral deflection data, representative of data shown in Figure 3 in the main text. (a) The raw data as recorded; (b) The data, overlaid with the data segments used for analysis (where tip is contacting the SA-CNF); (c) Zoom into the area marked by the dashed rectangle in [a], where the lack of sufficient tip-SA-CNF contact leads to the absence of a force curve (marked by arrow), and the resulting PFM signal is not taken into account (corresponding to dashed circles in Figure 3d).

S7 ND-PFM signal Reliability

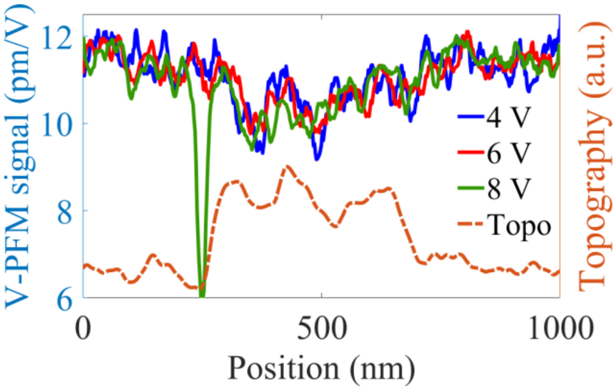

Figure S7. Vertical PFM signal obtained from the same site using different AC voltages. The calibrated measurements are in agreement. The 6V data is presented in main text Figure 3d (1). The negative peak is a measurement artefact.

S8. Additional measurements

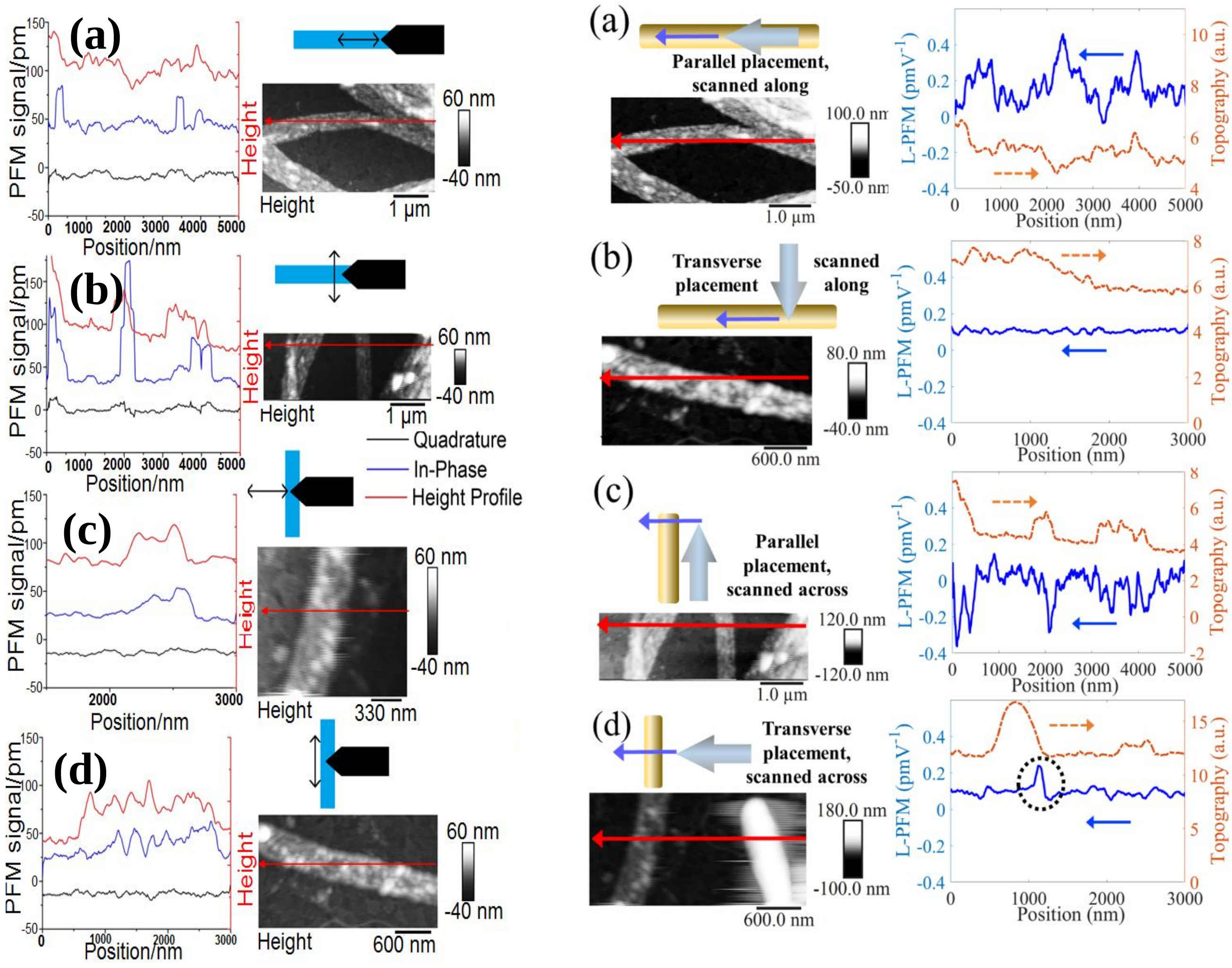

Figure S8. Vertical (left) and lateral (right) ND-PFM signals obtained without prior KPFM. Topographic images with a red arrow indicate the position and direction of the scan. Inset diagrams indicate the cantilever orientation and scan direction relative to the nanofiber. Height profiles are not to scale and are for guide to the eye.

S9. SA-CNF Topography

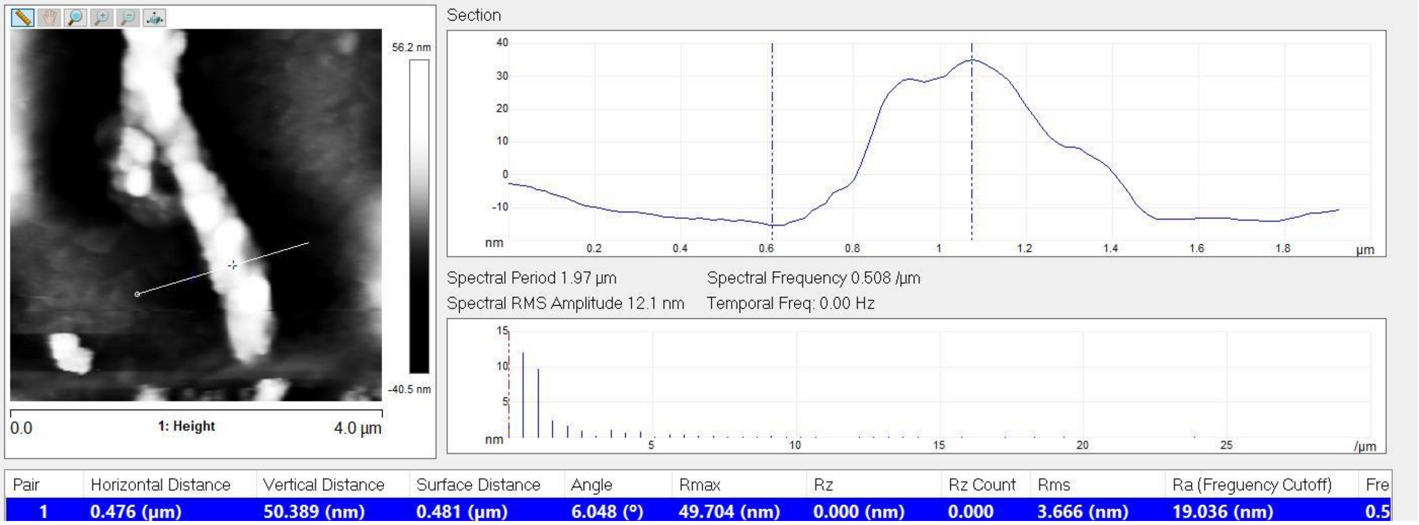

Figure S9. Topography cross section the SA-CNF shown in Figure 3b (main manuscript). Although the nano-pores are 200 nm in diameter, the measured height is 50 nm or less, while the width is 400-500 nm. This could be related to a tube-like structure collapsing.

S10. COMSOL piezoelectric coupling matrix

As mention above (section S1), the piezoelectric matrix of the monoclinic structure is effectively degenerated on wood. However, it is reasonable that highly oriented structures recover some of the coefficients. Therefore, we have used the following matrix to simulate the piezoelectric response in COMSOL (values in pm/V) inspired by Ref. [27]. These value are rough representations of the matrix form and **are not based** on experimental or theoretical data, since quantitative measurements generally deal with wood, or attribute measurements to the degenerate matrix. The model is shown below

$$d_{ij} = \begin{vmatrix} 0 & 0 & 0 & 5 & -10 & 0 \\ 0 & 0 & 0 & 10 & 5 & 0 \\ 10 & 5 & -10 & 0 & 0 & -5 \end{vmatrix}$$

We have also simulated structure with a "wood" like matrix, as follows

$$d_{ij} = \begin{vmatrix} 0 & 0 & 0 & 10 & 0 & 0 \\ 0 & 0 & 0 & 0 & -10 & 0 \\ 0 & 0 & 0 & 0 & 0 & 0 \end{vmatrix}$$

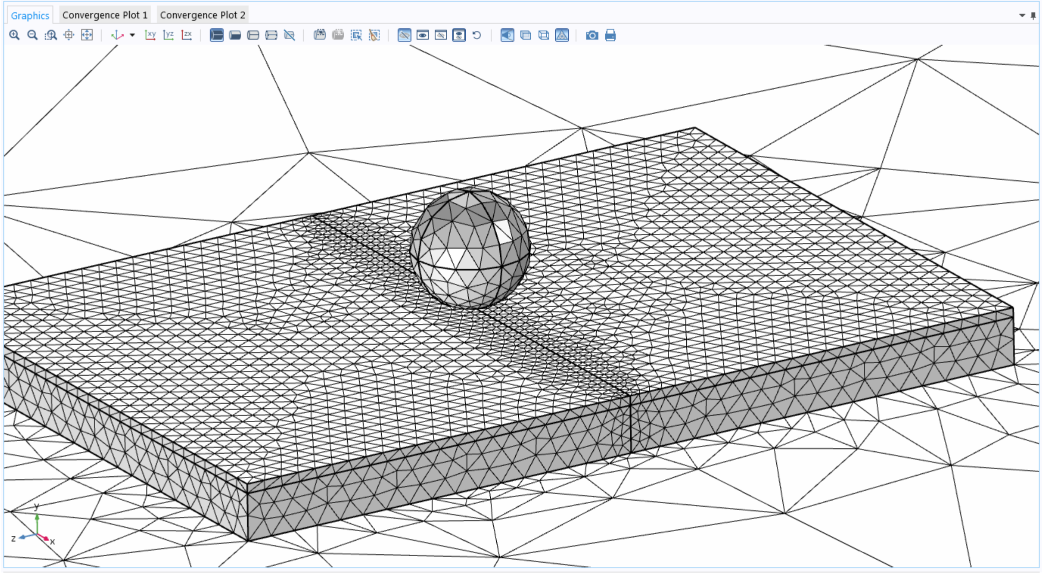

Figure S10. Screen capture of the COMSOL PFM model – the bilayer is clamped to the substrate underneath, while the sphere moves across the x-axis simulating an AFM tip apex.

References

(1) Moon, R.J.; Martini, A.; Nairn, J.; Simonsen, J.; Youngblood, J. Cellulose Nanomaterials Review: Structure, Properties and Nanocomposites. *Chem. Soc. Rev.* **2011**, *40*, 3941-3994.

(2) Fukada, E. Piezoelectricity as a Fundamental Property of Wood. *Wood Sci. Technol*. **1968**, *2*, 299-307.

(3) Fukada, E. Piezoelectricity of Wood. *J. Phys. Soc. Japan* **1955**, *10*, 149-154.

(4) Rajala, S.; Siponkoski, T.; Sarlin, E.; Mettänen, M.; Vuoriluoto, M.; Pammo, A.; Juuti, J.; Rojas, O.J.; Franssila, S.; Tuukkanen, S. Cellulose Nanofibril Film as a Piezoelectric Sensor Material. *ACS Appl. Mater. Interfaces* **2016**, *8*, 15607−15614.

(5) Frka-Petesic, B.; Jean, B.; Heux, L. First Experimental Evidence of a Giant Permanent Electric-Dipole Moment in Cellulose Nanocrystals, *Europhys. Lett.* **2014**, *107*, 28006 (5 pages)-

(6) Jungk, T.; Hoffmann, A.;Soergel, E. Consequences of the Background in Piezoresponse Force Microscopy on the Imaging of Ferroelectric Domain Structures," *J. Microsc.*, **2007**, *227*, 72–

78.

(7) Kim, S.; Seol, D.; Lu, X.; M. Alexe, and Y. Kim, "Electrostatic-Free Piezoresponse Force Microscopy," *Sci. Rep.*, vol. 7, p. 41657, 2017..

(8) Peter, F.;Rüdiger, A.;Waser, R.; Szot, K.;Reichenberg, B. Comparison of In-Plane and Out-of-Plane Optical Amplification in AFM Measurements, *Rev. Sci. Instrum.*, **2005**, *76*, 46101 (3 pages)

(9) Chen, W. S.; Yu, H. P.; Liu, Y. X.; Chen, P.; Zhang, M. X.; and Hai, Y. F.Individualization of Cellulose Nanofibers from Wood using High-Intensity Ultrasonication Combined with Chemical Pretreatments. *Carbohydr. Polym.* **2011**, *83,* 1804–1811.

(10) Fu, G.; He, A.; Jin, Y.; Cheng, Q.; Song, J. Fabrication of Hollow Silica Nanorods using Nanocrystalline Cellulose as Templates. *BioResources* **2012**, *7*, 2319–2329.

(11) Kumar, A.; Negi, Y. S.; Bhardwaj, N. K.; Choudhary, V. Synthesis and Characterization of Cellulose Nanocrystals / PVA Based Bionanocomposite. *Adv. Mater. Lett.* **2013**, *4*, 626–631.

(12) Kumar, A., Negi, Y. S., Choudhary, V.; Bhardwaj, N. K. Characterization of Cellulose Nanocrystals Produced by Acid-Hydrolysis from Sugarcane Bagasse as Agro-Waste. *J. Mater. Phys. Chem.* **2014**, *2*, 1–8.

(13) Liu, H.; Liu, D.; Yao, F.;Wu, Q. Fabrication and Properties of Transparent Polymethylmethacrylate/Cellulose Nanocrystals Composites. *Bioresour. Technol.* **2010**, *101*, 5685–5692.

(14) Man, Z.; Muhammad, N.; Sarwono, A.; Bustam, M.A.; Kumar, M.V.; Rafiq, S.Preparation of Cellulose Nanocrystals Using an Ionic Liquid. *J. Polym. Environ.* **2011**, 19, 726–731.

(15) Teixeira, E. de M.; Bondancia, T. J.; Teodoro, K. B. R.; Corrêa, A. C.; Marconcini, J. M.; Mattoso, L. H. C. Sugarcane Bagasse Whiskers: Extraction and Characterizations. *Ind. Crops*

Prod. **2011**, *33*, 63–66.

(16) Segal, L.; Creely, J. J.; Martin, A. E.; Conrad, C. M. An Empirical Method for Estimating the Degree of Crystallinity of Native Cellulose Using the X-Ray Diffractometer. *Text. Res. J.* **1959**, *29*, 786–794.

(17) Ahtee, M.; Paakkari, T.; Puikkonen, K.; Hattula, T. Crystallinity Measurements on Thermomechanical Pulp by X-ray Diffraction. *Pap. ja puu.* **1980**, *62*, 167.

(18) Weimer, P. J.; Hackney, J. M.;French, A. D. Effects of Chemical Treatments and Heating on the Crystallinity of Celluloses and their Implications for Evaluating the Effect of Crystallinity on Cellulose Biodegradation. Biotechnol. Bioeng. 1995, 48, 169–178.

(19) Savitzky, A.; Golay, M. J. E. Smoothing and Differentiation of Data by Simplified Least Squares Procedures. Anal. Chem. 1964, 36, 1627–1639.

(20) Diddens, I.; Murphy, B.; Krisch, M.; Müller, M. Anisotropic Elastic Properties of Cellulose Measured Using Inelastic X-ray Scattering, Macromolecules, 2008, 41, 9755–9759.

(21) Matsuo, M.; Sawatari, C.; Iwai, Y.; Ozaki, F. Effect of Orientation Distribution and Crystallinity on the Measurement by X-ray Diffraction of the Crystal Lattice Moduli of Cellulose I and II," Macromolecules, 1990, 23, 3266–3275.

(22) Sakurada, I.; Ito, T.; Nakamae, K. Elastic Moduli of Polymer Crystals for the Chain Axial Direction, *Die Makromol. Chemie*, **1964**, *75*, 1–10.

(23) Sakurada, I.; Nukushina, Y.; Ito, T. Experimental Determination of the Elastic Modulus of Crystalline Regions in Oriented Polymers, J. Polym. Sci., 1962, 57, 651–660.

(24) Lahiji, R. R.; Xu, X.; Reifenberger, R.; Raman, A.; Rudie, A.; Moon, R. J. Atomic Force Microscopy Characterization of Cellulose Nanocrystals, Langmuir, 2010, 26, 4480–4488.

(25) Hoeger, I.; Rojas, O.J.; Efimenko, K.; Velev, O.D.; Kelley, S.S. Ultrathin Film Coatings of

Aligned Cellulose Nanocrystals from a Convective-Shear Assembly System and their Surface Mechanical Properties, Soft Matter, 2011, 7, 1957, 2011.

(26) Jiang, M.; DeMass, S. N.; Economy, D. R.; Shackleton, T.; Kitchens, C. L. Formation of Highly Oriented Cellulose Nanocrystal Films by Spin Coating Film from Aqueous Suspensions, J. Renew. Mater. 2016, 4, 377–387.

(27) Zou, W. N.;Tang, C. X.; Pan, E. Symmetry types of the piezoelectric tensor and their identification. Proc. R. *Soc. A*, 2013, 469(2155), 20120755.
